# Remote cerebellar hemorrhage due to ventriculoperitoneal shunt in an infant: a case report

**DOI:** 10.1186/1752-1947-6-222

**Published:** 2012-07-30

**Authors:** Rakan Bokhari, Saleh Baeesa

**Affiliations:** 1 Division of Neurosurgery, Faculty of Medicine, King Abdulaziz University, PO Box 80215, Jeddah, 21589, Saudi Arabia

## Abstract

**Introduction:**

Cerebellar hemorrhage remote from the operative site is an unpredictable and rare complication in neurosurgery, with reported rates of morbidity and mortality in the literature of 8.4% and 7.8%, respectively. The range of procedures associated with remote cerebellar hemorrhage is diverse and includes both supratentorial and spinal procedures that entail significant cerebral spinal fluid loss or resection of supratentorial content. We present here the first documented case of remote cerebellar hemorrhage after controlled supratentorial cerebral spinal fluid drainage by ventriculoperitoneal shunt, and discuss the proposed pathophysiology and treatment.

**Case presentation:**

We present the case of a four-month-old Saudi Arabian male baby who presented with progressive symptoms and signs of congenital hydrocephalus. An uneventful ventriculoperitoneal shunting was performed with our patient recovering smoothly in the immediate postoperative period. On the next day, he had frequent episodes of vomiting and became lethargic. An urgent computed tomography scan of his brain revealed mild ventricular decompression and unexpected cerebellar hemorrhage. The infant was put under close observation, with marked spontaneous improvement over 48 hours and complete resolution of the hemorrhage on a follow-up computed tomography brain scan two weeks later. On regular outpatient visits at one, three and twelve months, he had no neurological deficit.

**Conclusion:**

Remote cerebellar hemorrhage is a complication that remains enigmatic in terms of both the underlying mechanism and clinical behavior. Our case revealed that the risk factors identified in the literature are not sufficient in predicting patients at risk of developing remote cerebellar hemorrhage. Our report also adds to the growing body of evidence challenging the currently accepted hypothesis explaining the pathomechanism of remote cerebellar hemorrhage. It thereby remains an unpredictable hazard that requires further study and increased awareness, as many cases in the literature are incidental findings.

## Introduction

Cerebellar hemorrhage remote from the operative site, referred to as remote cerebellar hemorrhage (RCH), is a morbid complication rarely seen in neurosurgery, with a reported incidence of 0.08% to 0.6% after supratentorial craniotomies [1,2]. Since its first description by Yasargil and Yonekawa in 1977 [3], this complication has received increased attention from the neurosurgical community. To date, more than 164 cases have been documented after supratentorial surgeries, not counting those occurring after spinal procedures [1,4]. Our understanding of this complication, its pathophysiology and risk factors is as limited as this phenomenon is rare. This poses a source of concern to the practicing neurosurgeon, as it is not without morbidity or mortality (up to 8.4% and 7.8%, respectively) [1]. Without a complete understanding of this phenomenon, we are unable to identify those at risk or decide the optimum line of management.

We present a case that adds to the growing body of evidence challenging the currently accepted theory explaining RCH. This case report is, to the best of our knowledge, also the first to document RCH as a direct consequence of the insertion of a properly functioning ventriculoperitoneal shunt (VPS). We review the literature for the proposed risk factors and mechanisms underlying this disease and contrast it to our case.

## Case presentation

A four-month-old Saudi Arabian male baby was presented to our neurosurgical clinics with a progressive increase in head size for four weeks. He was the first child for an unrelated couple and the outcome of an uneventful pregnancy. An antenatal ultrasound at week 34 of gestation reported mild ventriculomegaly. Nevertheless, he was delivered at full term with spontaneous vaginal delivery. At birth, his head circumference was in the 90^th^ percentile, without clinical evidence of increased intracranial pressure, and his general examination was within normal limits. His mother brought him because of vomiting and her concern with the rate of increase in his head size over his second month of life.

**Figure 1:**
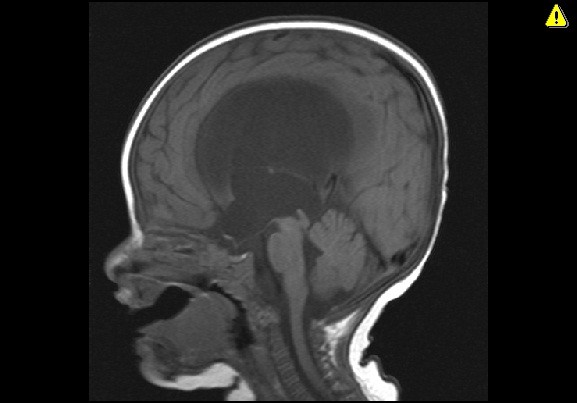
Sagittal T1-weighted magnetic resonance image demonstrating obstructive hydrocephalus due to congenital aqueductal stenosis.

On examination, our patient was alert and active with a normal physical examination, apart from ‘setting-sun’ eyes. His head circumference exceeded the 90^th^ percentile and he had a full anterior fontanel measuring 2 × 3cm in diameter. A cranial magnetic resonance imaging (MRI) scan demonstrated obstructive hydrocephalus due to congenital aqueductal stenosis (Figure 1). His routine laboratory tests, including a complete blood count, electrolytes, renal and coagulation profiles, were within normal limits. He underwent an uneventful endoscopic third ventriculostomy and was discharged home after 48 hours in good condition.

**Figure 2:**
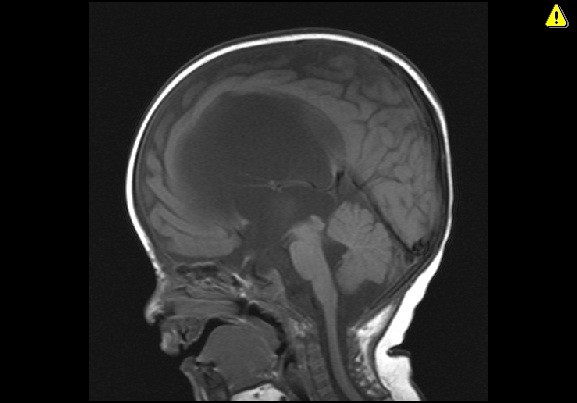
**Sagittal T1-weighted magnetic resonance image four weeks after the endoscopic third ventriculostomy.** Demonstrates unchanged ventricular size despite patent ventriculostomy

**Figure 3:**
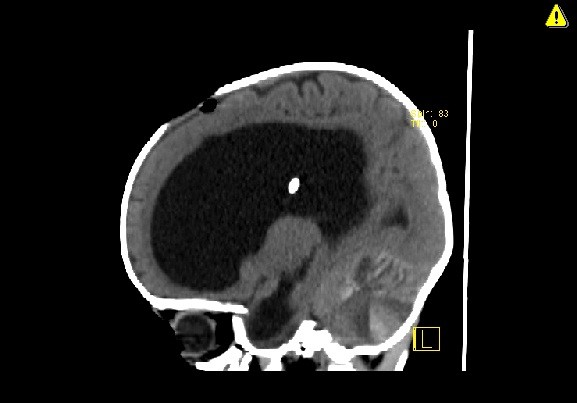
Sagittal computed tomography scan demonstrating the extent of the subcortical and intraparenchymal cerebellar hemorrhage. Note the classic zebra sign.

**Figure 4:**
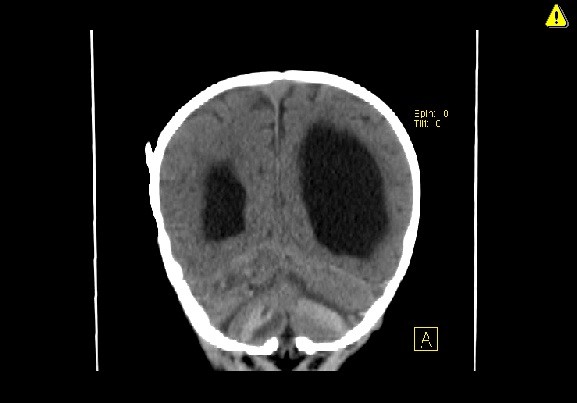
Coronal computed tomography scan demonstrating bilateral cerebellar hemorrhages.

**Figure 5:**
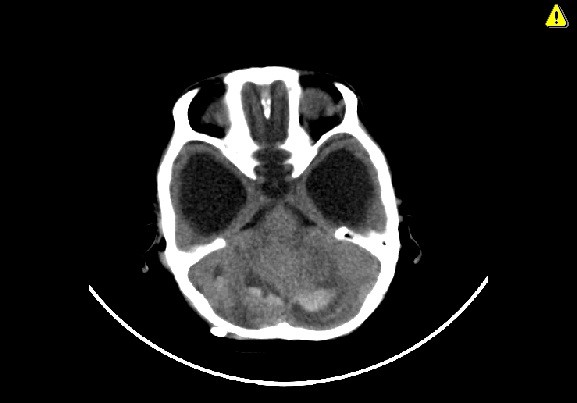
Axial computed tomography scan demonstrating bilateral acute cerebellar hemorrhage.

**Figure 6:**
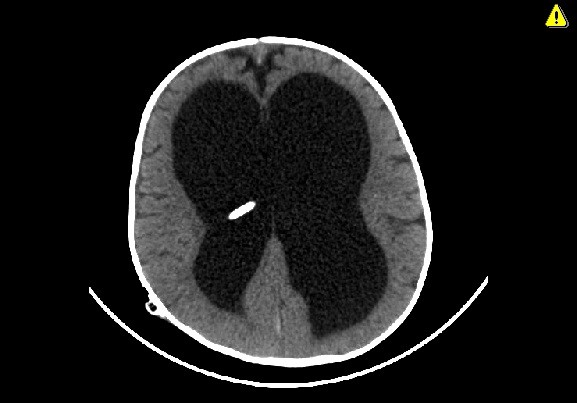
Axial computed tomography scan revealing no supratentorial intraparenchymal or intraventricular hemorrhage related to the catheter insertion.

A follow-up after four weeks revealed recurrence of vomiting and increasing head circumference with a full anterior fontanel. He was admitted and a repeat MRI scan revealed a patent third ventriculostomy with no significant changes in the size of the ventricles (Figure 2). He had a medium pressure VPS (P.S. Medical, Medtronic, Minneapolis, USA,) inserted. The procedure was carried out in the routine fashion and a ventricular catheter was introduced through a right parieto-occipital burr hole. Adequate placement was confirmed by the drainage of clear cerebral spinal fluid (CSF) under pressure, with care taken to drain less than 5cm^3^ to send for a cell count, chemistry and culture. The peritoneal catheter was inserted into the peritoneal cavity using a percutaneous technique. The procedure was uneventful, and our patient was extubated after surgery and started oral feeding after four hours. Eighteen hours later, he became lethargic with difficulty feeding and vomiting, despite a soft anterior fontanel. An emergency cranial computed tomography (CT) scan demonstrated a bilateral hyperdensity signal in his cerebellar hemispheres, representing hemorrhages (Figure 3, 4 and 5). There was no change in the size of the ventricles, and the ventricular catheter was in good position with no associated intraventricular hemorrhage (Figure 6).

Repeat laboratory blood tests revealed no coagulopathy. He was observed and eventually improved and resumed oral feeding within 48 hours and was discharged two days later. A follow-up CT scan after two weeks demonstrated complete resolution of the cerebellar hemorrhage. At one year follow-up, his neurological development was within normal limits with an adequately functioning VPS.

## Discussion

RCH is a rare neurosurgical complication believed to be a result of significant CSF hypovolemia. The list of procedures associated with this complication is extensive, with cases reported to occur after supratentorial, trans-sphenoidal and spine surgeries [2,4-6]. The common denominator in these cases is the occurrence of significant CSF drainage and hypovolemia. Many attempts have been made to identify the underlying pathophysiology but no consensus can be reached.

Although much controversy surrounds this entity, there are some aspects that appear to be agreed upon in the reviewed literature [1,4,6]. RCH is believed to be venous, as it is commonly bilateral with a predilection for the superior cerebellar surface and vermis, territories drained by the superior vermian veins [2]. Histopathologic examination of such cases also revealed the presence of venous infarction, implicating a venous rather than arterial source [6]. The other aspects agreed upon include the need for significant CSF hypovolemia to occur either during or most likely after the procedure. Furthermore, RCH is typically subarachnoid, with a variable intraparenchymal component that corresponds to the associated morbidity and mortality [2].

It is not clear which patients will go on to develop this complication with the literature implicating almost all procedures. It may be that many patients develop microbleeds, clinically silent forms of RCH. Only a small subset has certain perioperative and intrinsic risk factors that cause progression to the clinically manifest form. These include intraoperative and postoperative CSF loss, with negative pressure drains identified as one the strongest predictors of developing RCH [6,7]. Other factors suggested include perioperative hypertension, coagulopathies, platelet dysfunction (including perioperative aspirin administration), dehydration and, possibly, antibiotics [6,8,9].

The management of RCH follows the guidelines of cerebellar hematomas in general [6]. If no mass effect is noted, then a conservative line of management can be followed with good outcomes expected. The presence of hydrocephalus or significant mass effect, however, is an indication for intervention and a marker of poor prognosis [2]. The one aspect of management unique to this hemorrhage is the need to assess for either CSF over-drainage or the possible presence of an occult CSF leak. There have been advocates for correcting the CSF hypovolemia by infusing isotonic crystalloids but this has not been widely adopted by the neurosurgical community [7].

Theories in the literature attempting to explain the pathophysiology are numerous [1,2]. All converge to increase the transmural pressure gradient, stressing the infratentorial venous walls. Two general mechanisms to increase this gradient are presented in the literature. The first is by raising the venous pressure through impedance of the venous outflow. This may occur at several sites downstream, one is the jugular vein, which may be stretched against the cervical transverse processes as a result of certain head positions during surgery and thereby occluded [10]. Yoshida *et al*. suggested that another site of potential venous obstruction is at the superior vermian veins that course superiorly from the cerebellum [11]. As the cerebellum sags downwards from the CSF hypovolemia, they are stretched and kinked with resulting venous congestion. The other mechanism proposed to increase the transmural gradient, advocated by König *et al*., is by decreasing the ambient CSF pressure induced by over-drainage, this effectively results in venous hypertension [8].

The arguments for and against each theory are very well summarized in the review by Brockmann and Groden [2]. But it is our belief, as is the consensus in the literature, that the currently available theory that best explains the occurrence of RCH after both supratentorial and spinal procedures is the ‘cerebellar sag’ hypothesis [4,6,11]. It provides an explanation for the tendency of RCH to occur after procedures resulting in substantial CSF drainage, regardless of location and operative positioning.

This theory is not without its critics, as the end result of the cerebellar sag theory is obstruction of the vermian venous outflow. However, RCH is not a common occurrence in infratentorial surgeries involving the intentional occlusion of the supracerebellar veins [5]. Other weaknesses in this theory include the conspicuous absence of cerebellar edema expected with venous obstruction and, most recently, the theory’s failure to explain the occurrence of RCH after controlled CSF drainage, as in our case [12]. This is further evidence that we are yet to have one theory that can stand alone.

This theory, also, cannot alone explain why RCH caused by spinal procedures is associated with worse outcomes than a RCH after supratentorial craniotomies [1,2], so we present our hypothesis to explain this aspect. We believe that there are two forces that coexist in any given case of RCH: first, the force generated by a cerebellum migrating as it sags by virtue of gravity; and second, the force generated by the pressure gradient from the posterior fossa towards the site of CSF drainage. In spinal related RCH, these two forces, the cerebellar sag and the pressure gradient favoring caudal cerebellar migration, synergize. By comparison, in cranial related RCH, these forces counteract, as the gradient favors an upward migration that diminishes the net force, resulting in a milder hemorrhage and more benign course. We also found an interesting article linking intracranial hypotension to venous thrombosis, a mechanism not yet investigated for a role underlying RCH [13]. We do acknowledge that this would imply that antithrombotic agents would be protective and not a risk factor, as has been observed, but it may still have a role in impeding the venous outflow and thereby causing venous hypertension [4].

Our case challenges the believed prerequisite of massive CSF drainage for the development of RCH. Only one previous case of RCH has been reported as being caused by controlled CSF drainage [12]. In that case, RCH developed after the insertion of a lumboperitoneal shunt, while our case is the first to ascribe RCH to the supratentorial-controlled drainage of CSF in the form of a VPS. Our case does not offer any other explanation, as we are meticulous in our surgeries to drain no more than 5cm^3^ to prevent rapid expansion of the subdural space, and the shunt series showed no disconnection of the shunt components. We report a more benign course than that of the one with the lumboperitoneal shunt, which is in line with our belief of the role the gradient direction plays in the pathogenesis of RCH. We are not, however, the first to attribute RCH to a VPS, but in that other reported case, the cause of cerebellar bleeding was the well-intentioned but ill-advised compression of the reservoir over 200 times in 10 minutes by the patient’s relative, with an estimated forced drainage of 120 mL [14]. So, unlike our case, it supports the necessity of significant CSF hypovolemia in the pathophysiology of RCH.

Our case also questions the comprehensiveness of the risk factors mentioned in the literature and expands the affected demographic. Our patient was four months old, while the youngest previously reported patient was 14 years of age [1]. Our patient was normotensive for his age, and remained so throughout his admission. His coagulation profile and platelet count were normal, with no medications affecting hemostasis being administered. His intraoperative positioning was that of moderate extension and lateral rotation, the shunt had a functioning medium-pressure valve and he recovered without delay or deficit from anesthesia. The absence of surgical indications caused us to keep our patient under close observation for signs of deterioration. The reason why our patient developed this complication after a minimal amount of CSF had been drained, while many patients tolerate significant CSF hypovolemia well, indicates that we are still far from understanding the full scope of the pathogenesis and risk factors.

The presence of a shunt in a patient with RCH who is deteriorating would pose a challenging dilemma. The patient is dependent on the VPS, yet its presence risks the loss of the tamponading effect of CSF, allowing the hematoma to rapidly grow unabated [15]. This offers an attractive argument for the use of programmable shunts, offering the option of a slow, gradual lowering of CSF pressure and, should hemorrhage occur, pressure may be manipulated as needed.

## Conclusion

RCH is a poorly understood disease whose pathophysiology is still a matter of controversy. Our case adds to the growing body of evidence challenging the current prevailing theory’s requirement of substantial CSF drainage. It is the authors' belief that there is no single underlying theory that explains all RCH cases, and that there are in fact several subtypes, as evidenced by the varying morbidity observed between those caused by supratentorial and spinal procedures. Each of these subtypes is with their own underlying mechanisms that, along with certain patient- and procedure-related factors, converge to disrupt the infratentorial vascular bed. We would also like to stress the value of identifying those patients at risk of developing RCH, as we do believe that our patient was, for some reason, at a higher risk than usual to develop this complication, considering the minimal CSF drainage that triggered the event. Had we been able to label him as such, we probably would have elected to use a programmable shunt, and employed a gradual decrease of intracranial pressure postoperatively to obtain even slower and more strictly controlled CSF drainage over several days.

## Consent

Written informed consent was obtained from the patient’s father for publication of this case report and any accompanying images.

## Competing interests

The authors declare that they have no competing interests.

## Authors’ contributions

SB analyzed and interpreted the patient and radiologic data. RB performed the literature review and was a major contributor in writing the manuscript. Both authors read and approved the final manuscript.
